# Virtual reality content creation based on self-contained components in the e-learning domain: Re-using pattern-based vr content in different authoring toolkits

**DOI:** 10.1007/s11042-022-13362-5

**Published:** 2022-06-18

**Authors:** Robin Horst, Simon Gerstmeier, Ramtin Naraghi-Taghi-Off, Julian Wagner, Linda Rau, Ralf Dörner

**Affiliations:** RheinMain University of Applied Sciences, Kurt-Schumacher-Ring 18, 65197 Wiesbaden, Germany

**Keywords:** Virtual reality, E-Learning, Bite-sized learning, Authoring, Games engineering, Short virtual reality experiences, Virtual reality learning nuggets

## Abstract

In the context of e-learning, it is challenging to incorporate emerging technologies, such as alternate reality games or Virtual Reality (VR), within current learning trends. Microlearning is such a current trend. It divides large and complex chunks of content into small and elementary learning nuggets. These single self-contained nuggets are then composed to overarching lessons or courses. The concept of VR nuggets dovetails this educational trend. VR nuggets are standalone, self-contained, and rather short VR experiences that can be combined with other learning nuggets. By using initial implementations of VR nuggets, they can be used to let authors create VR earning content, for example, to let learners experience alternate realities. In this paper, we further investigate the VR nugget authoring concept and extent it. We introduce two novel authoring toolkits that rely on VR nuggets – one based on context-related module interaction (*CoNMoD*) and one based on visual scripting (*ViNS Tiles*). In two separate user studies, we examine the acceptance of the toolkits and compare them to existing authoring environments that also rely on VR nuggets but utilize different interface techniques. These studies’ results emphasize the importance of exchanging content between different established tools and indicate the acceptance of our tools regarding their hedonic and pragmatic qualities, also compared to existing tools from related work. As a conclusion, we propose an exchange format for VR nuggets that supports their reusability. It enables authors that use different toolkits to work together. They can utilize VR nuggets created with other toolkits and still use their own preferred toolkit. By means of an expert survey, we draw conclusions on technical aspects and a suitable platform to make VR nuggets available to the community. This survey indicates that potential authors would use such an exchange-approach for creating and presenting VR content and that they are willing to share their work and to contribute in a VR nugget authoring community.

## Introduction

Current emerging multimedia technologies enable users to experience alternate realities. Such technologies get more and more pervasive within the daily environments of people and these technologies become affordable and applicable concerning the costs and the ease of use. One important part of our everyday life that can benefit from adopting new technologies are lifelong learning activities. For example, mobile learning became an established tool within e-learning methodologies for continuous education, based on the ubiquity of mobile devices. In this context, emerging alternate reality technologies, such as Virtual (VR) and Augmented Reality (AR) are also expected to support current learning trends.

One current trend within this e-learning community is called microlearning [34]. It provides learners small and comprehensible pieces learning content, called *learning nuggets* [55]. It impacts the authoring of learning content, as it divides large and complex content in these small and elementary nuggets. Each learning nugget represents a short and self-contained learning unit. Learning nuggets are flexible and reusable, as these units can be integrated in learning sessions without a dependency on other learning nuggets. This trend was transferred to VR authoring methodology [31], referred to as *VR nuggets*. Additionally, it was proposed to create VR nuggets based on established design patterns of a domain. This makes VR nuggets reusable and the authoring approach highly sustainable.

Currently, there exist two authoring toolkits for VR nuggets [31, 33] that support non-VR-experts in creating content for such relatively short alternate reality experiences. But implementations of VR nuggets can only be used by the exact authoring toolkit that they were created with, since both rely on different representations of VR nuggets within their system design. To make the established pattern-based approach even more sustainable, already implemented VR nuggets must be made accessible for a whole community of educators that want to use VR nuggets rather than keeping them for private reuse only.

In this paper, we make the following contributions:

- We introduce two novel authoring toolkits aimed at domain expert authors who are not proficient with the technical foundations of alternate realities such as VR. Both rely on VR nugget implementations and enable authors to create multimedia VR content with them. The first allows authors to manipulate VR content through context-related direct interactions, whereas the second is based on a visual scripting approach. We discuss the underlying concepts, specifically the system designs and authoring processes.
- In two separate user studies, we evaluate our authoring toolkits and compare them. Additionally, we conduct a baseline comparison and contrast our toolkits to two existing authoring toolkits for VR nuggets by means of their hedonic and pragmatic qualities.
- To support the dissemination of VR nuggets and the usage of existing default VR nuggets as basis for novel adaptions, we also conceive a general data structure for VR nuggets and propose a VR nugget file format. Therefore, we make the abstract concept of VR nuggets persistent and enable authors to import and export VR nuggets between different authoring systems. By means of an additional expert survey, we draw further conclusions about the dissemination of VR content and the technical realization of a VR nugget exchange format.

This paper is organized as follows. The next section discusses related work. Thereafter, we state our previous efforts concerning VR nuggets. In Section 4, we describe our authoring toolkits and exchange format. In the fifth section, we depict the user studies that evaluate our authoring toolkits. Section 6 provides a conclusion and points out directions for future work.

## Related work

In this section, we describe related work about VR and microlearning, as well as pattern-based approaches and VR authoring.

### Virtual reality and microlearning

The effectiveness of using VR technology to support educational purposes in general is already widely examined and accepted throughout the last decades (e.g., [4, 5, 21, 24, 29, 53, 66, 67]). VR is a suitable medium to present learning content in a vivid and engaging way [54], whereas exclusively text-based activities can lead students to boredom relatively fast [68]. A view on the use of VR-related technologies within the field of microlearning shows work about mobile e-learning and Augmented Reality (AR) [51] technology. Beutner et al. [7] investigates micro units to deliver content to learners on their mobile phones. Souza and Amaral [59] provide a conceptual model how micro content can be used within mobile virtual environments. Both approaches bring small and self-contained content on mobile devices. Beaudin et al. [6] explore the use of AR technology to transfer small pieces of knowledge but do not refer to microlearning methodology specifically. Davies et al. [13] integrate 360^∘^ video VR into small group sessions within natural time constraints of university teaching sessions. Again, they use the technology in a nugget-like way, but do not specifically refer to this educational paradigm.

Finally, there are different established media for the usage within microlearning methodology [37]: ‘Reference Volumes’, ‘Books’, ‘Scientific Journals’, ‘Libraries of Working Papers’, ‘Images’, ‘Video Clips’, ‘Sound and Voice Recordings’, ‘Graphical Displays’ and ‘Electronic Mails’. In their study report, Job and Ogalo [37] pointed out that the newer media among them, such as mails and videos, were rated more important for microlearning than others. VR or other degrees of alternate realities were not mentioned in their study, which emphasizes that there is a lack of work that deals with integrating VR within this particular learning trend.

The recent existence of this identified research gap is also supported by current work (e.g., [1, 2, 49, 58]. Not only do current research efforts conclude that future online learning should by bite-sized [2], but the work by Ahmad [2] also points out very recent desire and opportunities for delivering bite-sized digital content during the COVID 19 Pandemic and its accompanying remote emergency teaching. After current work by Manning et al. [49], also cognitive load is reduced using bite-sized digital content. They also conclude that the design and development of digital learning content, such as VR content, could benefit. Concerning VR and related fields, a recent study by Alshehri [1] points out that Microlearning methodologies would benefit from the use of such novel technologies. However, he also concludes that due to the state of the art, AR technology should be favoured when planning Microlearning unity. This is because of technical barriers that exist concerning the authoring and usage of complex VR technologies in comparison to the more established and lightweight AR technologies (e.g., smartphones). However, the author still credits that VR technology, apart from existing technical issues, would be valuable for Microlearning purposes, also due to its higher immersion compared to AR. At last, a recent study with E-Learning experts [58] concludes that, after the assessment of the experts, that the form of microlearning content will be combined with new technologies such as virtual reality, augmented reality, and the Internet of Things and that this is very desirable after the experts. However, this study only expresses a desire rather than reflecting the current state. In conclusion, recent work underlines both the importance and the currency of investigating in VR technologies and their application within the field of Microlearning.

### Virtual reality and authoring

Apart from laying the technical foundation of a VR system, authoring describes the process of filling in or updating content to such VR systems.

Even though research in field of VR authoring has been conducted throughout the last decades (e.g., [9, 15, 60]), it still involves many challenges.

Pioneering work in the field of VR and AR authoring by Bierbaum et al. [8] and Schmalstieg [57] presents VRJuggler and Studierstube respectively. Both are frameworks for developing VR (VRJuggler) and AR (Studierstube) systems. VRJuggler offers a runtime environment that abstracts technical details, such as program code. In hiding these circumstances, it encourages designers to focus on the content of applications. VRJuggler allows changing content aspects at runtime, so that results can be viewed immediately, and authors benefit from the core idea of what-you-see-is-what-you-get (WYSIWYG) interfaces. In contrast, the Studierstube is an authoring system for AR applications. It also serves as a presentation system for the resulting AR applications. Both systems do not focus on supporting experts of a specific domain, but they are multi-purpose systems and provide basic technologies to create VR and AR applications.

VR authoring challenges are difficult to overcome for domain experts that are novel to VR or computer science in general. Usually, programming skills are required to maintain the content of an existing VR system. Additionally, the authoring for learning (and specifically microlearning) systems poses challenges [10, 40], as well. They not only concern educational experts, that have to overcome technical barriers, but they also apply for developers of learning systems, that must familiarize with common concepts and procedures of the educational domain. By including domain-typical approaches within VR development, the challenging communication process between system developers and domain experts can be reduced. Work by Dörner et al. [14] describes an authoring workflow that integrates domain experts within their AMIRE framework. This framework can be used to create Mixed Reality (MR) [51] systems and content.

Besides the involvement of domain experts, such as educational experts, there exist different types of interface technologies that can be used for VR authoring. One of them is visual programming. Figueroa et al. [20] and following work by Figueroa [19] investigates the use of a high-level visual programming language that supports rapid prototyping of virtual toolkits. They propose InTml, an application that includes three-dimensional geometry data (X3D) within their XML-based system. It also provides the ability to hide unnecessary information for design-oriented authors that do not want to change technical details, similar as in VRJuggler [8]. A visual editor is used to provide authors with support during visual programming. WYSIWYG aspects are not included, and Figueroa et al. describe that InTml [20] targets authors with profound knowledge about VR development.

There also exists work that includes design patterns for authoring purposes. With a view to alternate realities, there exists work about design patterns [47] and toolkits that incorporate design patterns [46, 62]. MacIntyre et al. [46] introduce DART, a multimedia toolkit for designers that facilitates AR development, whereas Tanriverdi and Jacob [62] introduce VRID, a system that utilizes communication patterns between objects to streamline the interface implementation process of VR development. MacWilliams et al. [47] investigate design patterns to be used for AR system development. They provide a catalogue of technical design patterns to describe AR systems. They use a pattern-description theme based on work by Gamma et al. [22]. When AR systems are built according to their patterns, the content for the resulting system must still be created separately.

Besides patterns, there exist authoring approaches and toolkits that utilize components within their systems to support the reusability of alternate realities. Klinker et al. [39] use a component-driven approach and include domain experts from the field of automotive design. They build an AR system based on recurring scenarios identified by the experts. The experts could use the resulting systems easily, since they were already familiar with the underlying scenarios of it. After all, patterns are already used beneficially for the creation of VR or related systems, but pattern-based approaches for content authoring are sparse. Still, studies have shown that domain experts can benefit from including established patterns of their domain in the creation process for VR systems.

Geijtenbeek et al. [23] propose *D-Flow* – a software tool supporting the development of clinical research and rehabilitation VR applications. Domain experts are provided with a graphical editor that uses visual programming to define feedback strategies for the subjects in VR. D-Flow’s system design consists of a layer for interfacing the hardware, a rendering system supporting multiple displays, and several modules. Each module performs low-level tasks as mentioned for other systems (e.g., network communication, interaction, logic, etc.). There also exist modules that provide domain-specific functionalities such as the *Human Body Model* module that ‘performs a biomechanical analysis to estimate and visualize muscle forces of a human subject in real-time’ (patent by Zohar and van den Bogert [69]). Modules can be utilized as building blocks to form high-level functionalities and build system functionalities for a specific use-case. The authors state that their system’s components themselves are not novel, but the combination of them with respect to the specific focus of the authoring tool on building VR applications for clinical research and rehabilitation. Overall, the work of Geijtenbeek et al. [23] demonstrates how domain-specific functionalities can be included within a VR authoring tool as independent and combinable components. However, applications built with D-Flow are still assembled from low-level components. This makes the tool one the one hand versatile (e.g., a Human Body Model can be utilized within different use-cases to simulate muscle forces, for example for rehabilitation, sports, or clinical applications) but on the other hand, authors still need expertise in developing VR systems to a degree that enables them to decide, for example, when to utilize collision detection, define particular value caps, or triggering global events. Furthermore, the provision of reusable components on the level of particular use-cases rather than entire domains is not investigated.

Carrozzino et al. [11] present *XVR Studio*, an integrated development environment utilizing a modular architecture, and *S3D*, a scripting language for VR development. S3D applications utilize a loop similar to the order of execution for event functions in game engines. For example, the function OnInit() initializes a project on startup, similar to Unity’s Awake() or Start() functions, and OnFrame() is called every frame of the loop to implement an application’s logic, similar to Unity’s Update() function. Furthermore, *S3D* wraps OpenGL functions so that high-level calls to low-level OpenGL code allow visual effects programming without modifying XVR Studio’s source code. XVR Studio provides different modules for developers, such as a script editor, a compiler, an FTP publishing module, and a text window for debugging purposes. For accelerating setting up a VR application, XVR provides authors with a wizard module that aims at guiding programmers through the setup process step-by-step, such as choosing the number of lights and cameras and their positions within the scene. Upon completion, the wizard creates a to-do list of sections that programmers can complete with code to fill, adjust, and finalize the VR application. Again, this component-based tool is intended to support the VR development tasks of programmers and does not shift competencies towards domain experts that may be involved in the overall authoring process. Besides providing a scripting language with VR-specific constructs and commands (e.g., for 3D animations, position-related sound effects, streaming, and UI), proficient programming knowledge is needed to use the tool.

Wang, Ijaz, and Calvo [64] state that the use of object-oriented application frameworks [18] that combine domain-specific design patterns within software components reduce the cost and improve the quality of domain-specific software and they propose a framework for developing VR experiences for the health domain. They utilize a definition of frameworks from Johnson [38], who defines this software technology as ‘reusable design of all or part of a system that is represented by a set of abstract classes and the way their instances interact’. However, similar to D-Flow [23], their system provides authors with low-level components to create a VR system. More abstract patterns, for example, on a level where a component may represent a specific use-case, are not investigated.

The work mentioned in this section uses 2D desktop PC technology, whereas immersive authoring relies on three-dimensional technologies. Lee et al. [44] use this term to describe their interface for AR authoring. Here, the target software itself serves as an interface for authoring. The same applies for immersive VR authoring. Lee et al. point out that the WYSIWYG-based ability to continuously test the content on actual AR devices supports the development workflow. More work on immersive authoring is conducted by Dunk et al. [16] and Jee et al. [36]. They propose a set of authoring interactions for CAVE-like VR systems and educational AR applications respectively. Takala [61] uses a game-engine-based VR toolkits (Unity [63]) and provides immersed authors predefined building blocks that can be used to ‘assemble’ a VR system. These blocks do not rely on specific patterns but illustrate an abstraction of VR system components.

Finally, reviewed work concerning VR authoring has shown that component-based systems can, on the one hand, present basic system functionalities as a service (physics engine, collision detection, etc.) that are utilized by expert authors within the system design. On the other hand, they can reflect blocks of content or classifying domain-specific content features to support non-expert authors. Concerning our subject, the consideration of the work supports the notion of involving domain-specific knowledge to support domain experts. However, none of the work utilizes content-related components of a higher level of abstraction than distinguishing 3D model parts semantically to support non-expert authors in finding relevant 3D models for composing 3D scenes. For example, on a more abstract level, a component could reflect an entire pre-made 3D scene classified by its intention (e.g., presenting a technical part such as a fuel cell). It remains open to explore such high-level abstractions of content-related VR components, for example, to investigate how they can be included and provided within a VR system and how such components can support domain expert authors in creating VR content. A further glance on related work we investigated during our literature research reveals that a plethora of recent work (e.g., [3, 25, 35, 41, 52, 56, 64]) sees potential and indicates a growing demand for investigating how the context of resulting VR applications can be taken into account within authoring processes and tools to support domain experts.

The use of patterns in VR-related and educational areas shows that pattern-based authoring approaches can support domain experts such as educators within their workflow. Identifying suitable educational design patterns for VR and integrating them in a VR authoring tool to be used by domain experts remain research gaps. Furthermore, the literature research has shown that most of the work focuses on utilizing patterns within approaches for design or programming experts (e.g., [39]). However, Ashtari et al. [3] identified that domain experts were able to use VR authoring tools (e.g., game engines) but did not feel confident that they could create a VR from scratch. Domain experts stated that they wanted to use existing examples as a starting point as they did not know where/how to start. Pattern-based approaches such as VR nuggets could help overcoming this challenge by providing the patterns as already implemented software that can be adapted during the authoring. Furthermore, related work indicates that context-related authoring that addresses goals of a particular domain can make the authoring of VR applications more accessible for domain experts and reduced the development time and effort [17, 23, 50, 64]. As a conclusion, domain-specific patterns should be included in VR authoring tools. None of the works enables domain expert authors to choose and select suitable patterns relating to VR content by themselves. Making the use of patterns transparent for domain experts during the authoring remains open for investigations. How are patterns provided to them? How is a pattern reflected within the system design of a VR authoring system for domain experts? How can a pattern address challenges on a narrow context (such as a use-case) rather than on a high-level context (e.g., providing rendering functionality)? Which content does a pattern provide, and how must such content be editable?

## Previous work on VR nuggets and VR-nugget-based authoring systems

This section states our preceding efforts in defining a learning-nugget-based concept for creating VR experiences as VR nuggets. Previous work about VR nuggets and their authoring builds the conceptual foundation for our work.

We conducted studies indicating that educational experts [32] and learners [30] could successfully use the integration of short learning-nugget-like VR experiences in-between other media. But the authoring of such VR experiences is still a challenge that prevents educators to use VR nuggets. The *VR nugget* concept dovetails the authoring paradigm of learning nuggets from the e-learning domain and transfers it to the area of VR authoring. We characterize the VR nugget concept by using relatively short VR experiences that can be placed between nuggets that are implemented with other media. Furthermore, we propose to use patterns as basis for VR nuggets.

The VR nugget concept divides a VR system into three components: (1) *VR nuggets*, (2) *functional coatings* and (3) the *nugget platform*. VR nuggets are the essential parts of the concept. They reflect elementary patterns from the application domain in small and self-contained VR experiences. They are provided for authors in the form of initial implementations that are already in an executable state.

**Fig. 1:**
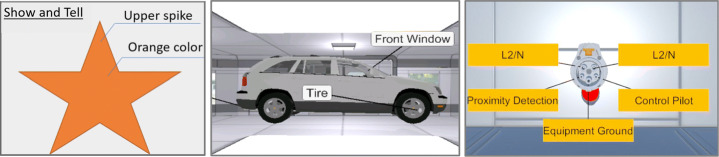
A conceptual illustration of the show and tell pattern (left) and two implementations of it (middle and right) [30, 31]

Conceptually, VR nuggets have parameters [31], based on their underlying patterns, that include the placeholder objects. An initial set of suitable microlearning patterns for VR nuggets is already identified [30]. In this paper, we will use as typical example their *show and tell* pattern (Fig. 1). It annotates a 3D object with callouts – short text strings with a spatial connection line – to give the learners information about its structure. For the show and tell pattern, authors must provide one main object that will be annotated and at least one callout that annotates it. The VR nugget system is designed to only allow one active VR nugget at a time. Domain experts can focus their attention on providing content to this one VR nugget instead of multiple nuggets simultaneously.

Functional coatings can be attached to VR nuggets to customize them without altering their original intention (e.g., a learning goal that the underlying pattern aims to fulfill). For example, when a VR nugget describes the parts that a specific car consists of, it might be sufficient that learners can walk around the car model and watch the annotated parts. A functional coating may extend the basic functionality, such as enabling learners to grab and rotate the parts or adding light beams to the VR scene that guide the learner’s view. But the VR nugget still informs learners about the composition of a car.

The nugget platform is the last component of the VR nugget system. It represents the runtime environment of VR nuggets and combines functionalities such as rendering, memory management and hardware control. Authors of VR nuggets do not have an interface to the nugget platform, so that technical details are hidden from them.

**Fig. 2:**
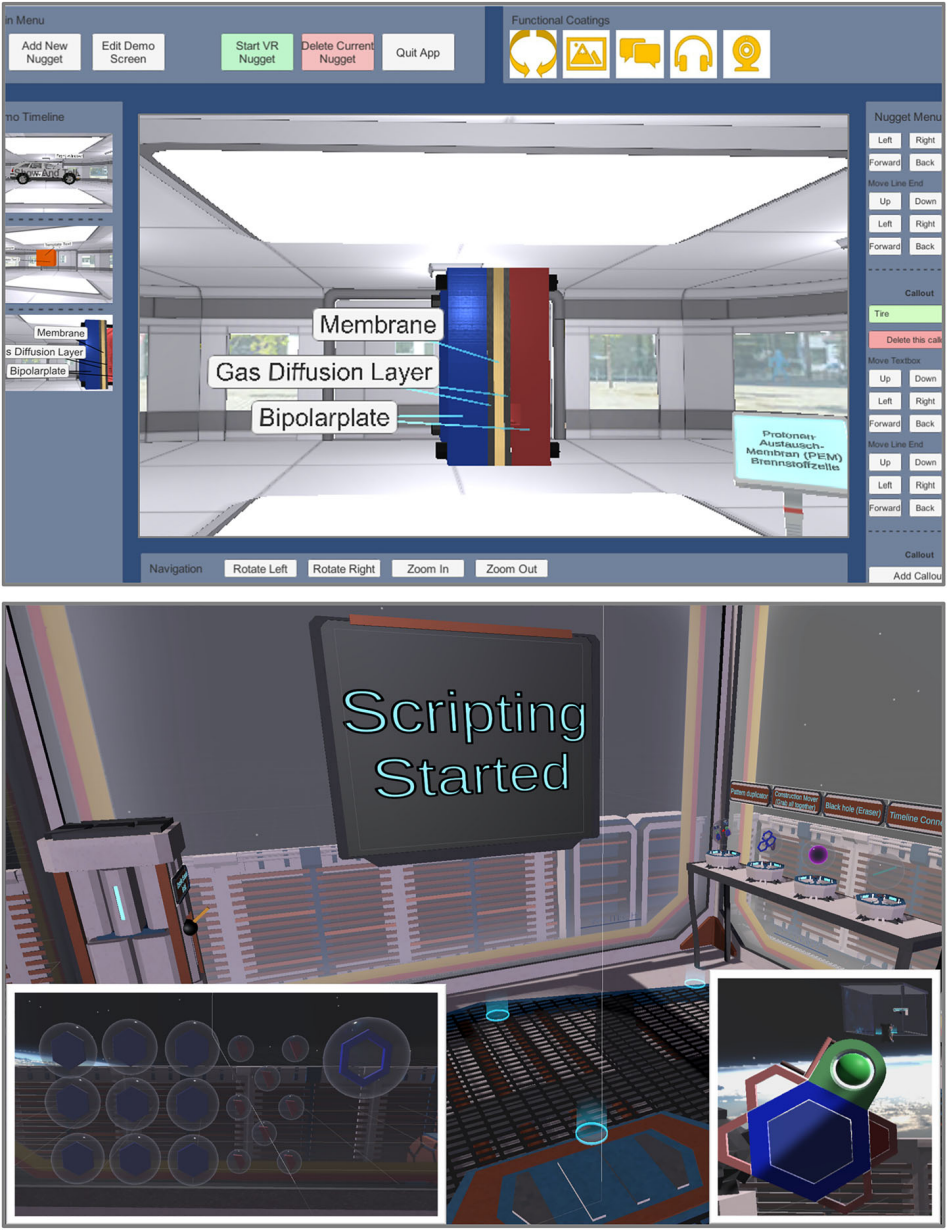
Left: The VR Forge authoring interface with a show and tell nugget active [31]. Right: The immersive VR nugget interface of IN Tiles [33]

Upon introducing VR nuggets conceptually and providing insights into the system design and the acceptance of educators and learners, we proposed two authoring systems meant for laypersons to create VR nuggets. There exist two authoring systems for VR nuggets – *VR Forge* [31] and *IN Tiles* [33]. VR Forge [31] is an authoring toolkit with a slideshow-inspired interface. It consists of six menus (Fig. 2 left). (1) The *main menu* (top left) provides system functionality, such as starting the VR nugget presentation or closing the application. (2) The *demo timeline* (left) provides an overview about linear arrangement of the VR nuggets. (3) The *current nugget menu* (middle) shows the currently selected VR nugget. It displays changes during runtime. (4) The *navigation menu* (bottom) enables authors to navigate through the current VR nugget implementation. (5) *Functional coatings* can be added from the top right menu. (6) The *nugget menu* (right) allows authors to replace the nugget parameters and adjust the scene objects.

**Fig. 3:**
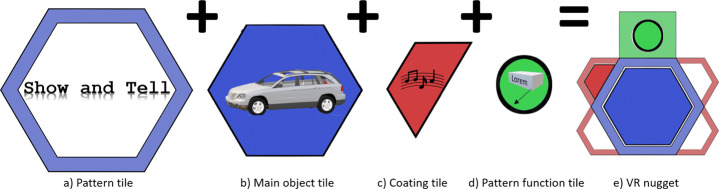
Conceptual illustration of the different tile affordances that IN tiles uses to let authors assemble VR nuggets

IN Tiles [33] provides an immersive authoring interface to create VR nuggets using VR hard- and software. The visual style of the interface is inspired by a high-tech look (Fig. 2 right). The interface utilizes tile-like shapes and provides visual programming interactions without code (visual scripting). These tiles represent affordances to be assembled. An assembled VR nugget is represented by 4 types of tiles (Fig. 3): (1) A single *pattern-tile* that determines the type of the selected VR nugget, (2) one *main-object-tiles*, (3) *coating-tiles* and (4) *pattern-function-tiles*. The latter control pattern-specific functionalities, such as the callouts of a show and tell nugget. 3D representations of these tiles are available within the virtual authoring environment.

The IN Tiles authoring approach is conceptually divided into three virtual rooms. (1) In the *authoring room*, the 3D tiles are assembled, and the content is loaded into the system. For each VR nugget representation, the authors can switch into separate (2) *editing rooms* that are associated to each VR nugget after assembling it. In editing rooms, the authors are placed within the corresponding scenes that a learner will be placed in when they use the VR nuggets. Fine-grained changes can be performed here, such as positioning virtual objects in 3D space. Authors also can switch from each editing to a (3) *demo room*, which hides all authoring-related aspects and lets them experience the VR nugget as the learners will experience it later. The IN Tiles authoring process is not strictly linear, so that authors can switch between the three rooms continuously.

Finally, IN Tiles uses immersive authoring [43, 44] technologies, which has certain system requirements regarding VR authoring applications and requires authors to perform also tiresome tasks such as text input to be performed using VR controllers. A transfer of IN Tiles’ authoring approaches to common desktop PC user interface technologies can deliver insights on what tasks of IN Tiles’ workflow might be more suitable to be performed without immersive technologies.

Both toolkits were evaluated. Their studies indicate that both toolkits can be uses successfully to author VR content and that IN Tiles is more oriented towards hedonic qualities. However, both rely on different data structure representations of VR nuggets. As a result, a VR nugget implemented in VR Forge cannot be used within IN Tiles and vice versa. Furthermore, domain expert authors are not enabled to include novel VR nuggets in an authoring system and investigated user interfaces do not include visual scripting approaches on more conventional desktop PC technology. This should be explored and compared to current VR nugget authoring tools to point out how an authoring approach that makes use of pattern-based VR applications in well-defined scope and targets non-expert authors should be designed.

## Novel VR nugget authoring toolkits and the VR nuggets exchange format

In this section we introduce two novel toolkits for authoring alternate realities and facilitate the creation and consumption VR learning experiences. Both tools rely on the concept of VR nuggets. We call the tools *CoNMoD* and *ViNS Tiles*. At the end of this section, we propose our VR nugget exchange format.

**Fig. 4:**
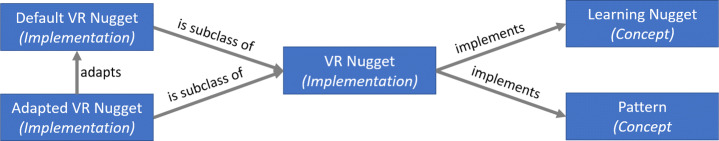
The relation of patterns, small and self-contained learning nuggets and classes of VR nuggets

Related work has shown that a VR-nugget-based authoring approach uses existing VR nuggets as basis for future adaptions with similar intention, so that the authors do not have to implement each VR nugget from scratch. In this work, we call these initial VR nuggets *default VR nuggets* and thereof adjusted VR nuggets *adapted VR nuggets*. Default VR nuggets comprise placeholder objects and predefined learner interactions with them. During the authoring process, authors simply exchange the placeholder objects with their own content. This ensures that a VR nugget remains always in an executable state. We illustrate the relation of a design pattern, learning nuggets, VR nuggets and these two sub-classes in Fig. 4.

### CoNMoD – co ntext-related n ugget mo dules with d irect content interaction

Within the CoNMoD authoring toolkit, we divide between three authoring roles: The *content author*, the *nugget author* and the *system author*. Content authors makes use of predefined default VR nuggets and exchanges the placeholder-content with their own content to form adapted VR nuggets. This role is supported by existing VR nugget authoring systems. However, one author of a VR nugget systems must initially create the default VR nuggets. We refer to this user role as the nugget author. The system author is the author who creates the overall authoring system and does not mandatory have to create default nuggets of a system. In existing systems, the responsibilities of creating the overall system and the default VR nuggets is the same author, so that they only divide classify between content authors and a system author that also create the default nuggets. However, it can be beneficial to enable authors that did not create the system to create novel default nuggets for content authors.

**Fig. 5:**
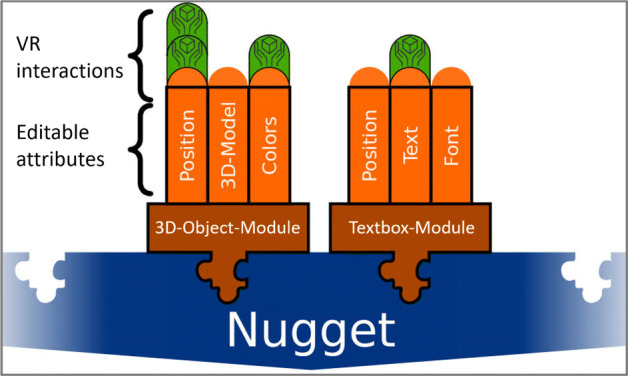
Conceptual VR nugget architecture for a show and tell pattern within the CoNMod toolkit

CoNMoD supports both content authors and nugget authors in addition to system authors. Content authors can use default VR nuggets with placeholder objects, whereas nugget authors can access detailed sub-structures of VR nuggets. Therefore, we divide VR nuggets into different *modules*. Modules reflect the parameters of the VR nugget concept that are replaced during the authoring process. For a show and tell pattern, a VR nugget consists of *object-modules* and *text-box-modules* (Fig. 5). CoNMoD provides different authoring interactions with the virtual content depending on which module is currently selected. These modules have *editable attributes*, such as a position, a color or a 3D model. Each attribute can be assigned as set of *VR interactions*. These interactions relate to the underlying attributes which again affect the underlying modules.

**Fig. 6:**
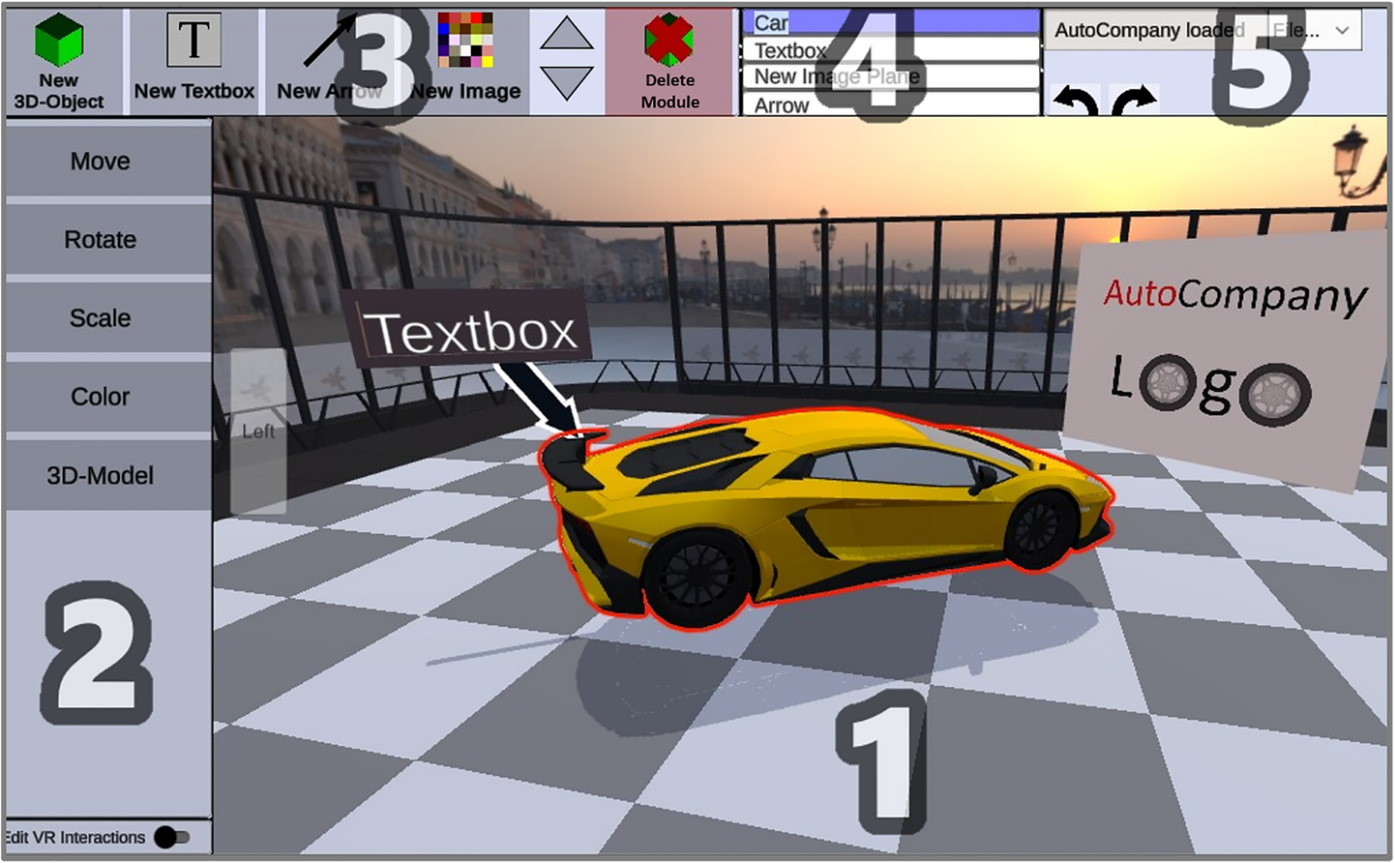
The context-related CoNMoD authoring interface which consists of five panels

The graphical user interface of CoNMod consists of five panels (Fig. 6). Users can directly interact with the content of the current active VR nugget by clicking on the virtual objects within the (1) *view port*. Each of these objects within the scene represents a module. The view port is always centered on the current active module, and rotation and zoom functionalities are provided so that authors can navigate around the modules. This can help authors to focus on editing the current module (centering) and can provide them an overview (rotation and zoom). Authoring interactions with these objects are provided within (2) the *context menu*. For example, Fig. 6 illustrates a case where the main model (car) is selected, which can be moved, rotated, scaled, colored or replaced by another 3D model. For most of these adjustments, the context menu only activates them, and the actual actions are performed directly within the view port. For example, when the ‘move’ action is activated within the context menu, a handle appears within the view port that can be dragged directly for moving the car object. The top left panel represents the (3) *creation menu* which provides possibilities to insert new modules to the scene. (4) The *overview menu* shows a simplified scene graph as a drop-down list. This scene graph is condensed to the modules of a VR nugget and can be used to find and select specific modules. It helps authors to get an overview of the active VR nugget in case it consists of many modules. The last panel represents the (5) *system menu* and provides meta-functions, such as undo, redo, switching between VR nuggets, loading and saving.

Inserting new modules to a nugget from the creation menu can alter the nugget to reflect a novel pattern or alter the intention of the underlying pattern. This feature can be used by nugget authors to change existing default or adapted nuggets into novel nuggets that can reflect novel pattern, as well. These can then be used as default nuggets in future authoring.

One specific module of the CoNMoD toolkit is the visual surrounding for a VR nugget. In the case of Fig. 6, we chose to provide a subtle coastline 360^∘^ image as a skybox for the VR nugget. This surrounding-module can be replaced similar to other placeholders. Apart from this skybox-based surrounding, we chose to limit the area where modules can be placed. We call this area the *VR stage*. The boundaries of the stage area are visualized by a fence that prevents learners from walking out of the desired area. During the authoring process, the VR stage is adjusted automatically. Authors can place their modules freely within the VR stage, and when they get close to the fence the stage expands seamlessly.

**Fig. 7:**
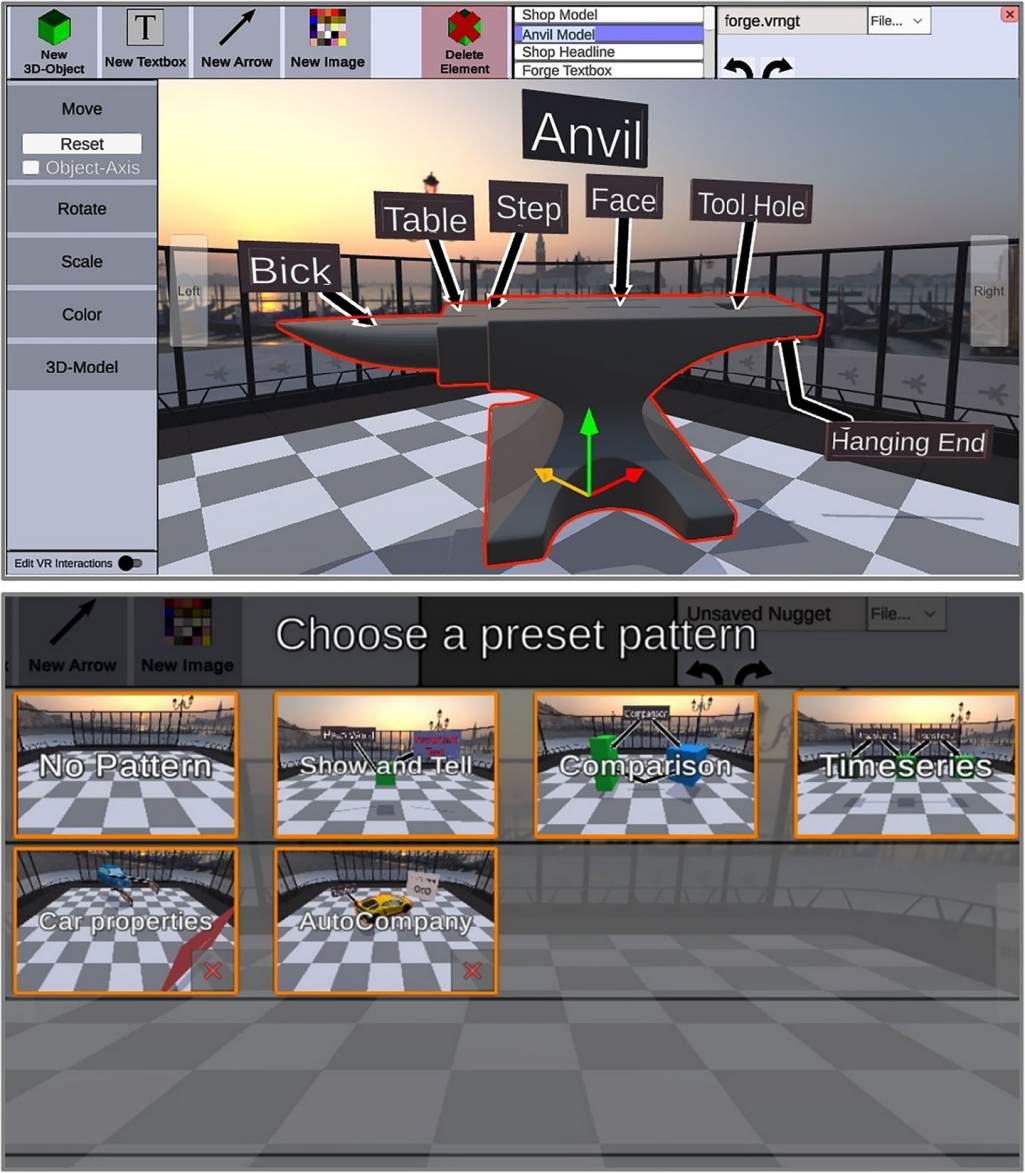
Left: An implementation of a show and tell pattern. Right: Pattern-selection screen

We provide authors a separate *pattern-selection screen* (Fig. 7 (right)) where they can choose between pattern. After deciding on one of them, a default VR nugget realization of the chosen pattern is provided. A realization of a show and tell pattern is illustrated in Fig. 7 (left).

For implementing CoNMoD, we use the Unity [63] game engine. The Virtual Reality Tool-kit (VRTK) [45] is used for the VR hardware connection. It has a simulator that can be run on a second screen and shows the VR nugget as learners would see it during the authoring. If a supported VR head-mounted display (HMD) is plugged into the desktop PC that runs the CoNMoD toolkit, changes can also be viewed directly within the HMD. This WYSIWYG feedback during runtime allows authors to perceive their changes to the virtual environment continuously throughout the creation process.

### ViNS Tiles – Vi sual N ugget S cripting Tiles

**Fig. 8:**
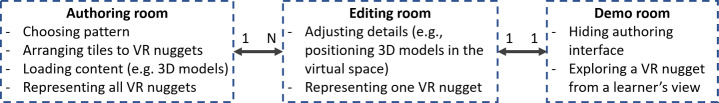
Relation and functionality of the three rooms that our visual scripting approach is divided into

ViNS Tiles is an authoring toolkit that builds on both the VR nugget concept and the IN Tiles toolkit described in the related work section. It transfers the immersive authoring approach from IN Tiles onto a desktop-PC-based setup and reflects the affordances and interactions within a 2D visual scripting environment. We also divide our authoring process of ViNS Tiles into three semantic spaces, called *rooms* (Fig. 8), and divide authoring interactions into: The *authoring room*, the *editing room* and the *demo room* (similar to IN Tiles [33]).

**Fig. 9:**
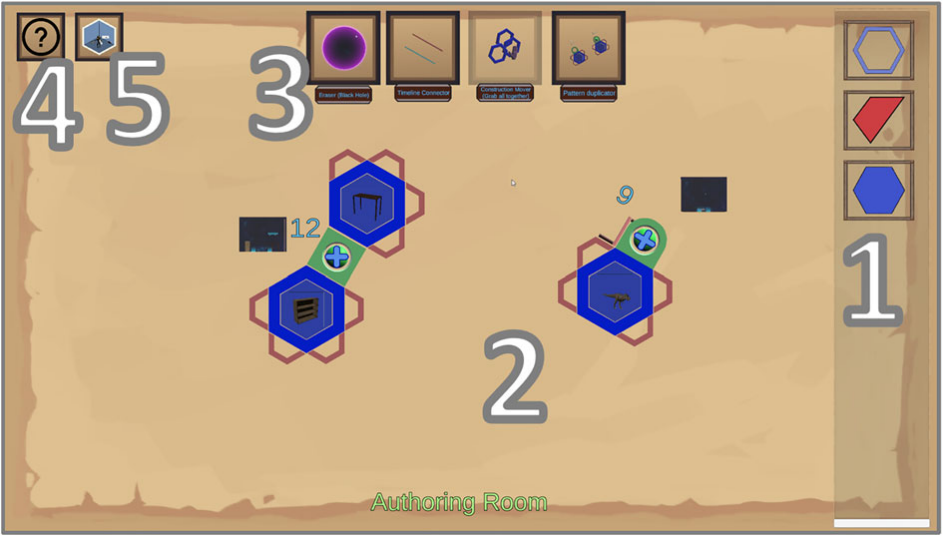
The ViNS Tiles interface of the authoring room which consists of five menus

The interface of the authoring room consists of five menus (Fig. 9). In the (1) *tiles menu*, authors can select different tiles. Hints regarding the functionality of the tiles are displayed there, as well. VR nuggets can consist of four different types of tiles (see Section 3). Tiles are spawned on the (2) *work bench menu*. The work bench can display several VR nuggets that are used within one curricular structure, such as a lesson. Upon spawning a pattern-tile, affordances for coating- and pattern-function-tiles are attached automatically. Therefore, each new VR nugget starts with a pattern-tile on the work bench. The tiles can be arranged and assembled to form VR nuggets by clicking and dragging. Upon spawning a main-object-tile, a placeholder 3D model is provided to ensure that VR nuggets are executable at all time. When the placeholder is exchanged by the author, a preview of the model is visualized on top of the main-object-tile so that authors can get an overview about the content directly at the work bench (Fig. 9). The (3) *tools menu* lets authors map their interactions to different authoring features. It can change the preselected *assembly tool* to a *delete tool*, a *connect tool* or a *copy tool*. Delete can be utilized to remove entire VR nuggets or single sub-tiles from the work bench. The connect tool is utilized to bring VR nuggets in a temporal relation. The copy tool duplicates entire VR nuggets on the work bench. Single elements from the duplicate can be substituted by new content to adapt an VR nugget based on the same pattern. This allows that similar VR nuggets do not have to be assembled from scratch. The (4) *FAQ menu* provides help functionality in the form of an FAQ document and an image-based tutorial. The (5) *switch menu* is utilized to switch between the three rooms of the authoring process.

**Fig. 10:**
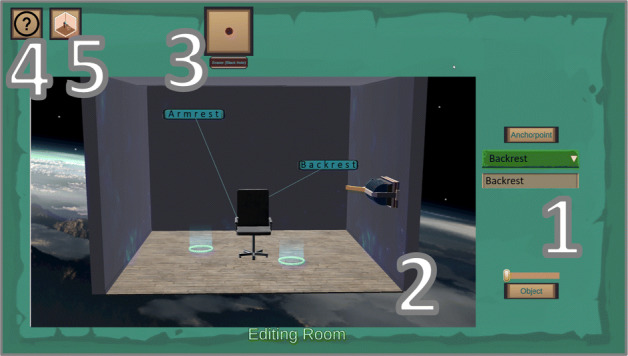
The ViNS Tiles interface of the editing room

The editing room consists of a similar menu setup (Fig. 10). It is composed of a (2) *work bench* area that shows a three-dimensional representation of the corresponding VR nugget, a (3) *tools menu* that allows switching to the delete tool, a (4) *FAQ menu* that provides help and a (5) *switch menu* that provides room switching functionality. The editing room differs in terms of the tiles menu, which is substituted by the (1) *attributes menu*. It provides functionalities to change attributes of the virtual content, such as moving, rotating or scaling the content, but also entering text for the callouts for show and tell. To support the same continuous testing activities as the original IN Tiles toolkit, we also provide a demo room experience for the authors (see Section 3). As for its immersive equivalent, the editing room can be turned into the demo room by hiding and disabling all above mentioned menus, except a button that switches back to the editing experience.

We refer to the tile-based visual assembly approach as *object focused*. All tiles are attached around the shape of the main-object-tile. Existing work on IN Tiles (Section 3) describes to support patterns that rely on exactly one main object, so that VR nuggets always are represented as in Fig. 3 (right). But there may exist patterns that rely on more than one main object. For example, a compare pattern [30], that visually compares two 3D models by connecting semantic aspects of the 3D models with lines. This pattern relies on two main objects and cannot be mapped to the visual affordances of the IN Tiles toolkit.

**Fig. 11:**
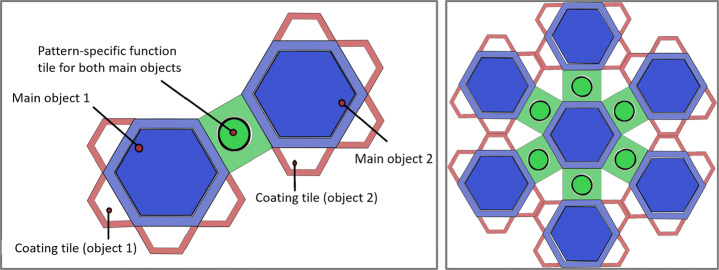
The extension of the tile-based concept of IN Tiles (Section 3) that supports more than one main object. Left: VR nugget representation with two main objects where pattern-specific functions are applied to both objects (e.g., comparison of two objects). Right: Seven main objects are used, where one object shares one specific function with every other object (e.g., comparison of one object with six other objects)

Besides providing a novel authoring interface for the tile-based approach, we extend the visual model so that VR nuggets can consist of more than one main object. In case of the compare pattern, the pattern-function-tiles (green dots within green rectangles) connect main objects. We use this semantic relation for the visual assembly model illustrated in Fig. 11 (left). Single pattern-tiles are still restricted to one main object that fills it, but we use more than one pattern-tile to represent VR nuggets with more than one main object. These pattern-tiles are directly connected by the pattern-function-tiles. This way, *multi-object patterns*, such as the compare pattern, are still represented as one coherent structure. This supports the clarity of the work bench menu and comply with the existing visual approach of IN Tiles. Figure 9 shows two assembled VR nuggets in the authoring room. One relies on a single main object (show and tell, right) and one on multiple main objects (compare, left).

Overall, the proposed hexagonal design for multi-object VR nuggets supports patterns that rely up to seven objects. At the example of the compare pattern, one object can be compared to aspects of up to six other objects (Fig. 11 right) – one connection at each side of the hexagonal pattern-tile. One could argue that our design also allows creating structures with more than seven main-object-tiles, for example, by attaching pattern-function-tiles to the outer pattern-tiles. However, this would not model a direct semantic relation between the middle main object and the novel outer objects, since the middle is only connected visually to the first six objects. In case of the coating tiles, our design does not limit the initial hexagonal design. On the contrary, due to coatings being not object-specific, but relate to an entire VR nugget, the proposed extension enables authors to use more than the initially proposed limitation of five coatings, since we provide coating affordances for each of the novel attached object tiles (Fig. 11).

We implemented the ViNS Tiles authoring toolkit using Unity [63] for the visual interface and the logic and VRTK [45] for defining the VR hardware interface. Again, the simulator is used to experience the VR scene independently of the VR hardware, but still enables authors to switch to the VR HMD within the demo room to experience the current VR nugget.

### VR nugget exchange format and dissemination

We proposed two novel authoring toolkits. Together with the two existing systems (VR Forge and IN Tiles, Section 3), this makes four systems that rely on the VR nugget concept. Despite using the same data-representation for the resulting short and self-contained VR experiences, each system internally uses a different data structure to represent a VR nugget. This restricts VR nuggets implemented in one of the systems to be executed by this exact system only.

#### Exchange format

**Fig. 12:**
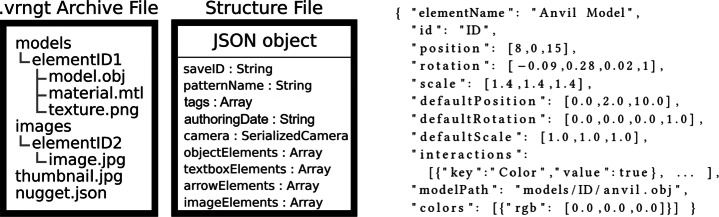
Left: .vrngt archive file structure. Middle: JSON file structure of a show and tell VR nugget. Right: Example JSON representation of a serialized object

We propose standards for a VR nugget file format to support cross-system deployment of VR nuggets. For the persistence definition of a VR nugget, we represent one VR nugget as one distinct VR nugget file with the ending ‘.vrngt’. Overarching courses of VR nuggets are then assembled with several VR nuggets loaded after another. The .vrngt is an archive file which we divide into four categories (Fig. 12 left). The first category (*models)* is related to 3D models, as they are basic elements of VR nuggets. The models-sub-folder within the archive consists of different sub-folders itself – one for each model used in the VR nugget. In each model-folder, necessary files regarding 3D models are located. For example, in case of an .obj-based model, there is a .obj, a material and a texture file. The second category is represented by a sub-folder, as well. *Images* contains images and videos that are used within the VR nugget – again, each in a separate sub-folder. We refer to the content of these first two categories (models and images) as *elements* within our exchange format. Each element has a randomly generated identifier (ID). Each ID only occurs once within one VR nugget so that it can be used for unique naming of the sub-folders of elements (Fig. 12 left). The third category of the VR nugget archive is covered by a single *thumbnail* image that can be utilized during the VR nugget selection process. It gives authors a preview of the VR nugget to facilitate the selection of VR nuggets for domain experts. At last, there is a *nugget.json* file.

The JSON file represents semantic relations and localization of the VR nugget content within a VR scene. The structure of the object that was used for serialization of a show and tell VR nugget is illustrated in Fig. 12 (middle). JSON was chosen over comparable formats, such as XML, because Unity’s Mono-based C# dialect is object-oriented, and the data structures can easily be parsed into an object notation such as used by JSON. The nugget.json file contains arrays of serialized elements and their metadata. It also contains VR-nugget-specific metadata that facilitate the dissemination of VR nuggets. We included the name of the underlying pattern, the authoring date and free text tags. This metadata can be used by authors to search for suitable patterns.

All instances of an element type are stored in an array that is dedicated to hold the serialized objects of this type. The objects hold the values of every attribute of the elements. Some attributes are shared by all element types such as position, rotation, scale and a name. The object in Fig. 12 (right) is an example of one serialized element (an anvil 3D model). This serialization contains all necessary information to load each element in the state that the VR nugget was saved by the authoring system. We chose to include default-values for attributes to provide authors to reset values to their original value during the authoring approach. The scene-graph information of the VR nugget is represented by its archive file structure. Individual files within this structure can be referenced by a relative file path because the imported files of the elements are saved in an archive. In Fig. 12 (right), the ‘modelPath’ field links the VR nugget element with the corresponding 3D geometry that is stored in the ‘anvil.obj’ file.

From a content author’s point of view, VR nuggets consist of placeholder objects to be exchanged during the authoring process. On a conceptual basis, we further divide between *exchangeable content* that authors actively have to provide (e.g., a 3D model that shall be annotated and text) and *generic content* (e.g., 3D text-box-panels and connection lines). This content is pattern-specific in terms of not every VR nugget makes use of it, but still generic in a way since it can be used by more than one VR nugget. Whereas exchangeable content is replaced by the authors, they are provided with the generic content by the authoring system, such as content relating to the function-tile concept of ViNS Tiles.

This characteristic can be included within our JSON structure, as well. A possible extension of the JSON object can be two lists that contain the IDs of either elements that are VR nugget parameters or pattern-specific content. These lists can be read by VR nugget authoring software and processed accordingly. At the example we used in the CoNMoD toolkit (Fig. 12 middle), we chose to allow nugget authors to insert novel exchangeable content freely during the authoring and did not include the lists in the structure. This provides authors the possibility to adjust VR nuggets to their convenience and make use of both exchangeable content and generic content. Furthermore, nugget authors that are already familiar with VR nuggets can use this feature to realize novel patterns in VR and save them as default VR nuggets to provide them for other authors for future authoring.

At last, a .vrngt file is loaded back into the system by processing the ‘nugget.json’ file and placing every element according to the saved coordinates and then importing the files from the archive. The archive is temporarily unpacked to be used in Unity. Then the element attributes are applied from the parsed values. For example, the colors of a 3D-model-element are stored in a simple RGB array. This can also be used to describe possible interactions with VR content for learners. VR-specific interactions are saved as a basic map of interactions to booleans, such as *can be grabbed* or *is touchable*. Every interaction that the author activates is mapped to true and all deactivated interaction to false. The actual realization of the interactions is handled by each distinct VR nugget authoring toolkit individually.

#### Database connection

**Fig. 13:**
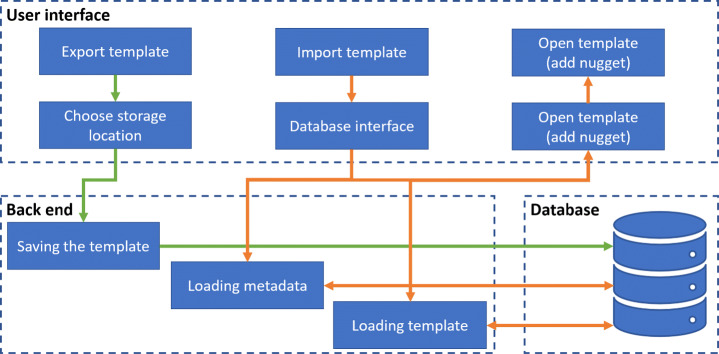
System architecture for loading (importing) and saving (exporting) procedures of VR nugget dissemination. Green connections denote the export and orange lines to the import of VR nuggets

The VR nuggets (.vrngt) files can now be stored locally but to create an exchange platform for VR nuggets, a connection from our game engine based authoring toolkits to a database is necessary. It supports making VR nuggets widely accessible for a community and can serve as central point of contact for sharing VR nuggets. In order for our Unity-based toolkits to establish the connection with a database, the Unity-internal interface for database access can be used. However, the purely Unity-internal conversion has a disadvantage. Parallelization of Unity accesses to external entities and especially databases is a non-trivial task for the game engine. Unity does not process such accesses parallel to the game loop, so that for the preparation of the previews, especially in the export phase, the user interface must be stopped, and the user must wait for the completion of the process. Furthermore, all conceivable extensions such as the creation of automated VR nugget proposals or synchronization with another database must be implemented by external components that access the database in parallel to Unity. This results in the necessity to block database access for individual components and leads to unwanted duplication of code. To avoid these challenges, the concept we developed is based on a connection model that separates the user interface and the processed components. This concept is shown in Fig. 13. In this design, the three central components are separated from each other: The *user interface*, the *back end* and the *database*.

The user interface provides users two functions: The export and the import of VR nuggets. If an export to a database is selected, the author must fill in data fields queried by the system and confirm the entries to complete the process. The import includes VR nugget previews that can consist of different information, ranging from fully textual descriptions of underlying patterns to preview images of the VR nuggets or a filter system. For example, VR nuggets can be filtered by means of user-ratings from authors that already utilized the proposed VR nugget.

The back end contains functions for preparing VR nuggets for the database and vice versa for Unity. It also stores the VR nuggets in the database. If a VR nugget is exported, necessary metadata that was not queried in the user interface can be generated automatically to facilitate the export-process for authors (e.g., using the date provided by the operating system as the date of creation). If the back end receives the information that an author wants to import a VR nugget, the metadata is loaded from the database, prepared for display in Unity and transferred to the selection mask of the user interface. After the author has selected a VR nugget, the actual content is loaded and transferred. This prevents all relevant VR nuggets from being loaded in full size, as this can result in performance losses when using many and/or large VR nuggets.

Our modular system structure supports that the back end can be extended, since the programming language and the frameworks used are freely selectable. This also enables a free choice of the database technology.

**Fig. 14:**
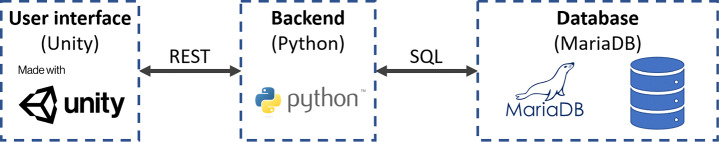
Tools that were used for implementing the dissemination system architecture

For implementing our system design, we used the tools illustrated in Fig. 14. The programming language Python is used for the back end. Since Python is a scripting language, we implemented all functions of the back end in one script and controlled them with different calls.

To enable a connection between the Unity user interface and the Python back end, that does not require the permanent execution of the back end script, the representational state transfer (REST) technology is used. To implement a REST service which communicates via http, a local Apache2 web server was utilized. A MariaDB SQL database is used as a database in our prototype. It is also locally installed and created with one user and a metadata-table with the fields defined as follows:

- Name – Contains the name of the VR nugget defined by the author. Since this field serves as the primary key of the table and thus for the unique identification of the VR nuggets, it must not be entered twice.
- Description – The description of the VR nugget in textual form (e.g., the tags from the exchange format, which were entered by the author).
- Author – Represents the name of the user who exported the VR nugget.
- Date – The day of export, generated by the back end.
- VR nugget – Contains the exchange format archive (.vrngt).

Finally, it the size of the assets (e.g., 3D models) that are used within adapted VR nuggets is crucial for the proposed client-server architecture. For example, downloading a VR nugget including a 3D model with a large size may require a fast internet connection and a large amount of storage on the operating system’s device. Our authoring process for layperson authors assumes that such 3D models are either already present, created with external tools (e.g., CAD modelling tools, Blender, or 3D Max), or obtained from external sources for assets such as the Unity Asset Store, Turbosquid, or Thingiverse. However, 3D models modeled for other purposes than VR or downloaded from the internet may be not optimized, even though the mentioned online sources provide the option to search for 3D with a low polygon count. To counteract the mentioned issues, a semi-automated pre-processing pipeline with external tools could be used that is also operable by laypersons, such as Autodesk 3ds Max’s *Batch ProOptimizer*. However, game engine technologies such Unity that we used also provide optimization possibilities to reduce the size of 3D models that are not built for realtime graphics systems. For example, existing Unity plug-ins from the official asset store such as *Mesh Simplify*, *Runtime Mesh Simplifier*, or *Runtime Mesh Operator* could be included within the export process.

## Evaluation

We conducted an expert survey to draw conclusions on the dissemination of VR nuggets. Furthermore, for evaluating the alternate realities authoring toolkits, we conducted two separate user studies that evaluate the user experience of authors with the proposed tools. The reports of these studies were used to compare both toolkits to each other and to the existing VR nugget authoring systems.

### Expert survey for disseminating VR nuggets

We conducted an anonymous expert survey with 17 participants that are professionally related to computer science and presentation methodology to explore further requirements for the dissemination of VR nuggets. They were particularly recruited from IT-related companies with interest but not experience in VR authoring technologies. In this survey, participants were asked about their opinion on both the technical integration of VR nuggets and the behavior of authors regarding the dissemination of them. We divided the 17 items (I1-I17) of the survey into four categories (C1-C4). All items were translated into the native language of our participants. For C1-C3, the participants were asked to rate the importance of the item’s aspect, given a 7-point scale (0=superfluous, 1=not important, 2=rather not important, 3=indifferent, 4=rather important, 5=important, 6=essential). The questions of C4 could be answered on a 3-point scale (0=never, 1=rarely, 2=often). For all four aspects, we provided the participants the possibility to comment on them within free text fields.

- Usability of the dissemination-approach
  - The dissemination functionality must be directly visible in the authoring interface (not in a sub-menu).
  - As much metadata as possible should be generated automatically (non-automatically generated information must be entered manually).
  - It is important to me that I can also store the VR nuggets locally.
    - VR nugget previews which are displayed in the import-mask, should:
    - contain a graphical preview of the content
    - contain a description
    - include the name of the author
    - contain the date of the last modification
- Functional scope of the system – Possible functions are:
  - Individual suggestions of VR nuggets based on previously loaded data
  - Display of the available VR nuggets in the order of an evaluation system
  - Release of VR nuggets only for certain users
  - Importing individual components of the VR nuggets
  - Import function for textures and animations
- Technology of the system – The dissemination-approach requires program elements outside Unity.
  - Platform compatibility (Windows, macOS, Linux)
  - Possibility of using a local database as a mirror (permanent access)
  - Free choice of database (e.g., MySQL, MongoDB, Neo4J)
- Author behavior
  - Would you use imported VR nuggets as basis to create new VR experiences?
  - Would you make your VR experiences available to other users (export public)?

**Fig. 15:**
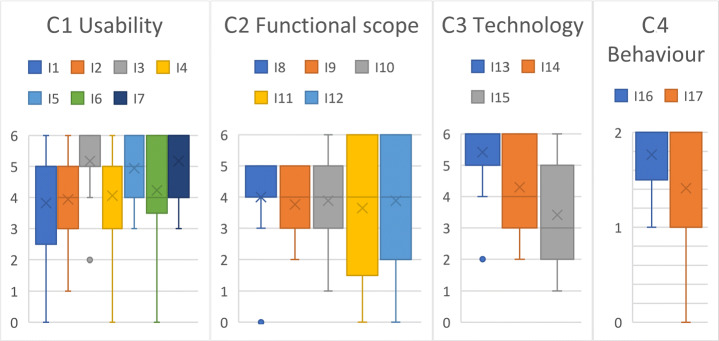
Outcome of the expert survey on the dissemination with n = 17 participants as box-whisker plots

We conducted Wilcoxon Signed-Rank tests [65] on item each compared to the hypothetical neutral value of the scale (3). We used a threshold for statistical significance of 5%. While the absolute mean values for each item were higher than 3, the test did not confirm a statistically significant difference at I1, I11, I12 and I15. All other items had a significantly higher value than the hypothetical mean. The following p-values were calculated: I1: *p* = 0.1096; I2: *p* = 0.03486; I3: *p* = 0.00042; I4: *p* = 0.0278; I4: *p* = 0.00044; I5: *p* = 0.00044; I6: *p* = 0.01352; I7: *p* = 0.00064; I8: *p* = 0.01552; I9: *p* = 0.0151; I10: *p* = 0.03; I11: *p* = 0.17384; I12: *p* = 0.09296; I13: *p* = 0.00042; I14: *p* = 0.00528; I15: *p* = 0.25428; I16: *p* = 0.00148; I15: *p* = 0.0391. Figure 15 illustrates the outcome of our survey with a box-whisker plot for each category C1-C4.

Regarding the usability (C1), our participants stated that a desired VR nugget dissemination must provide an interface within the authoring toolkit that enables authors to access the dissemination functionality directly (I1).

Our survey report also indicates that the possibility to save VR nuggets locally on the machine (I3) and not only online was rated as highly important. Furthermore, our participants indicated to include all proposed metadata about the VR nuggets within the selection screen (I4-I7) but assessed the creation/update date as the most important information for selecting VR nuggets. The participants of our survey rated the importance of items for the functional scope section (C2) rather scattered. They would like the dissemination system to suggest VR nuggets based on their previously used VR nuggets (I8) and that the system can sort VR nuggets according to a rating system (I9). The majority also expressed that the system should have a user management, so that authors can restrict to share their VR nuggets with specific users (I10), for example, when the VR nugget contains confidential content. Regarding the technology of the dissemination system (C3), our participants indicated that platform compatibility (I13) is a highly desired feature. This also applies to the use of a local database (I14). The ratings of the two items relating to user behavior (C4) indicate that our participants would use such a dissemination-based authoring system frequently within their profession (I16) and that most of them are thoroughly willing to share their VR nuggets for other users (I17). Only one of the participants stated that a public export would not be an option.

### Authoring toolkit user studies

Aside from the online expert survey, we conducted two distinct user studies to evaluate CoNMoD and ViNS Tiles – one study for each authoring toolkit. Based on these reports, we compare CoNMoD and ViNS Tiles to each other and also to the existing two VR nugget authoring toolkits (VR Forge [31] and IN Tiles [33]) regarding their hedonic and pragmatic qualities. We analyzed four aspects:

- **Ease of use**: Is the creation of VR nuggets manageable for authors?
- **Workflow**: Do the authors know which actions to perform next at any time during the authoring?
- **Clarity**: Can the authors handle the creation of multiple self-contained VR nugget applications or are there challenges to overview a larger project?
- **Efficiency**: Are the authors satisfied with their results in relation to the time spent during the authoring?
- **Product character** [26]: How do the participants perceive the tools regarding hedonic and pragmatic qualities?

We formulated the following null hypotheses H1_0_-H5_0_ and alternative hypotheses H1_a_-H5_a_ based on the mentioned aspects. With these hypotheses, we evaluate how the authoring toolkits differ and how future authoring toolkits should be designed.

- CoNMoD and ViNS Tiles are equally easy to use.
- CoNMoD and ViNS Tiles are not equally easy to use.
- CoNMoD’s and ViNS Tiles’s workflows are equally understandable.
- CoNMoD’s and ViNS Tiles’s workflows are not equally understandable.
- CoNMoD’s and ViNS Tiles’s user interfaces present the handling of multiple VR nuggets equally clear.
- CoNMoD’s and ViNS Tiles’s user interfaces do not present the handling of multiple VR nuggets equally clear.
- Authors are equally well satisfied with the efficiency of CoNMoD and ViNS Tiles.
- Authors are not equally well satisfied with the efficiency of CoNMoD and ViNS Tiles.
- The product character of CoNMoD, ViNS Tiles, VR Forge, and IN Tiles is perceived equally well.
- The product character of CoNMoD, ViNS Tiles, VR Forge, and IN Tiles is not perceived equally well.

Each user study involved 15 unpaid, voluntary participants (CoNMoD: 4 females, aged between 20 and 57 years with Ø 31.2 and SD 12.10; ViNS Tiles: 6 females, aged between 23 and 59 years with Ø 31.07 and SD 10.98). Our population came from a variety of sources related to educational purposes, such as departments from companies professionally related to presentation methodology. We also included participants from the wider university’s surrounding such as employees with teaching duties and student tutors.

On average, our participants classified themselves as laymen in the fields of VR and 3D software with mediocre knowledge of educational practices. Regarding technical experience with VR and 3D software, three participants in each study stated to have profound knowledge. The studies were conducted as follows: Participants were welcomed and informed about the VR nugget concept in a brief oral description. They were introduced to the software and then guided towards tasks they should accomplish with the authoring toolkits.

Concerning ViNS’s Tiles study, the first task asked the participants to adapt two default VR nuggets based on a different pattern each. 3D objects, texts, and functional coatings had to be used. In the second task, the participants had to adjust specific aspects of the already adapted VR nuggets from the first task, such as repositioning and removing callouts. In the last task, they were asked to adapt another default VR nugget of their choice, then make two copies of them and bring them in a temporal order using the meta authoring tools. In the end, they were asked to test the VR nuggets they had adapted from a learner’s point of view with the attached Oculus Rift S HMD.

Concerning the CoNMoD’s study, the first task asked the participants to create a show and tell VR nugget by inserting modules into the empty scene. Content such as 3D objects and texts were given. In the second task, the participants had to adapt a given show and tell default VR nugget. During the adaption, it was requested to delete and adjust provided callouts from the default VR nugget. After the adaption, the VR nugget had to be saved. In the third task, the participants were asked to utilize their saved VR nugget as a start to create a novel default VR nugget by assigning attribute extensions to the modules. In the last task, they were asked to apply their knowledge to a given use-case, and adapt a chronological sequence to illustrate the oil processing to fuel within a VR experience.

After the studies, the participants were asked to fill out a questionnaire that consisted of custom questions (Qc1-Qc17 for CoNMoD and Qv1-Qv15 for ViNS Tiles), each relating to a certain aspect A1-A4. Concerning the product character [26] (A5), both questionnaires included an abbreviated version of the AttrakDiff [27, 28] questionnaire as an established measure to draw conclusions on pragmatic qualities such usability and hedonic qualities. The questionnaires were translated into our participants’ native language. The 17 questions Qc1-Qc17 for evaluating CoNMoD and 15 questions Qv1-Qv15 concerning ViNS Tiles are listed in Table 1:

**Table 1:** The 17 questions Qc1-Qc17 for evaluating CoNMoD and 15 questions Qv1-Qv15 concerning ViNS Tiles

Questions	Associated aspect
CoNMoD questions	
Qc1 How did you find the usage of the tools (move, text, etc.)?	A1
Qc2 How easy did you find it to place, rotate and scale the elements in the room?	A1
Qc3 How easy did you find it to link the text boxes to the desired points on the object?	A1
Qc4 How free did you feel regarding the camera control?	A1
Qc5 Did you have enough space for your content in the 3D room?	A3
Qc6 How complete do you consider the set of available VR nugget elements (3D model, text box, arrow, images etc.)?	A1
Qc7 How understandable was the switching between the tools?	A3
Qc8 How did you feel about the freedom to place and edit the VR nugget elements?	A2
Qc9 How understandable was the workflow from an empty room to a finished VR nugget implementation?	A3
Qc10 How did you like the workflow of creating your own default VR nuggets and then creating several adaptions from it?	A2
Qc11 How important do you find the possibility to use and create your own default VR nuggets?	A2
Qc12 How understandable was the difference between the tools and the adjustment of VR interactions?	A3
Qc13 What do you think of the modular system with the nugget elements as components?	A2
Qc14 What do you think of the time required to create a nugget with the VR nugget editor?	A4
Qc15 How exactly do the nuggets you created reflect the information you wanted to present?	A4
Qc16 How well can you replicate the same results with different software?	A4
Qc17 How much time would it take with this other software?	A4
ViNS Tiles questions	
Qv1 How well were you able to distinguish the three different rooms from each other?	A3
Qv2 Was the functionality of the individual rooms clearly recognizable?	A3
Qv3 How did the change between the three rooms affect you?	A2
Qv4 How easy did the interaction with the building blocks work?	A2
Qv5 How well did the interaction with the tools (delete, move, connect and copy) work?	A1
Qv6 How helpful were the tools for solving your tasks?	A2
Qv7 How much did the shapes of the building blocks help you to understand the structure of a VR nugget?	A2
Qv8 Did you feel you had enough space to create your VR nuggets?	A3
Qv9 How easy did you find it to link the callouts to the desired points on the object?	A1
Qv10 How well did you get an overview of the assembled construct (VR nuggets) in the virtual world?	A3
Qv11 How understandable was the workflow for the creation of the VR nuggets for you?	A2
Qv12 How satisfied were you with the results of your implemented VR nuggets?	A4
Qv13 Did the creation of the VR nuggets take an acceptable amount of time for you?	A4
Qv14 How well can you replicate the same results with different software?	A4
Qv15 How much time would it take with this other software?	A4

We used a 7-point semantic differential scale to capture the data from the questionnaire items stated above, except for Qc8, where ‘3’ indicated the most positive value. Participants could indicate that they had too little or too much freedom. It was mapped to fit the scale of the other items. The studies were performed within a time frame of 1 hour per participant.

#### Analysis of the results

**Fig. 16:**
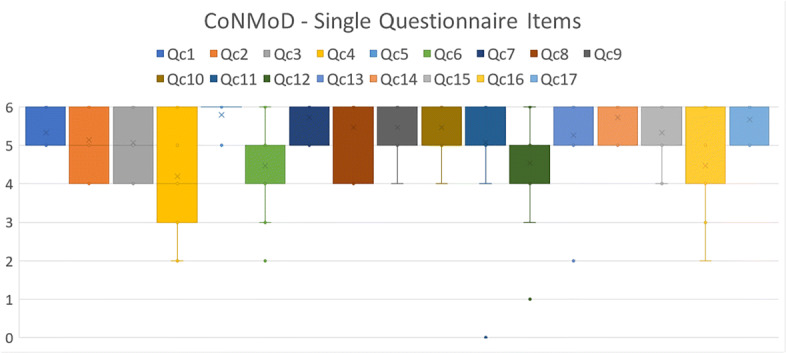
Descriptive statistics of the single items Qc1-Qc17 for CoNMoD using box-whiskers plots

Figure 16 represents the value distributions of the single items Qc1-Qc17 for CoNMoD in a descriptive statistic. It shows that the medians of the box-whiskers plots of all single questions lie above the value of 4 Questions Qc4 and Qc16 have the largest deviations, ranging from values of 6 to 2. Qc5 presents the narrowest box-whiskers spectrum consisting of the single value of 6. Outliers can be observed at Qc5, Qc6, Qc11, Qc12 and Qc13. The outliers at Qc11 and Qc12 are the lowest values of all items with a value of 0 and 1 respectively

**Fig. 17:**
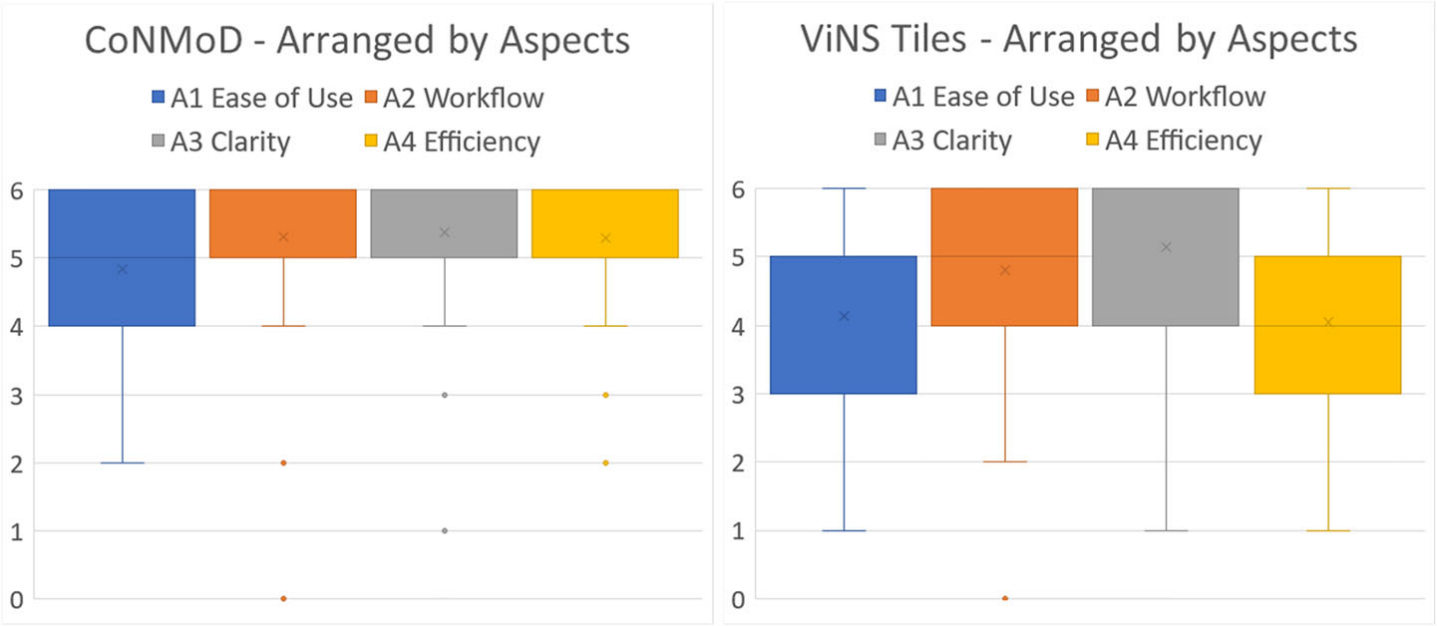
Descriptive statistics of the item values for CoNMoD (left) and ViNS Tiles (right) aggregated by the four aspects A1-A4 using box-whiskers plots

The outcome of the single items for CoNMoD were aggregated to the four aspects A1-A4. Results are illustrated in Fig. 17 (left). Mean values for A2-A4 lie above 5 and the box plots range from 4 to 6. They present outliers ranging from 0 to 3. A1 does not show outliers. It has a mean value between 4 and 5 and the plot ranges from 2 to 6.

**Fig. 18:**
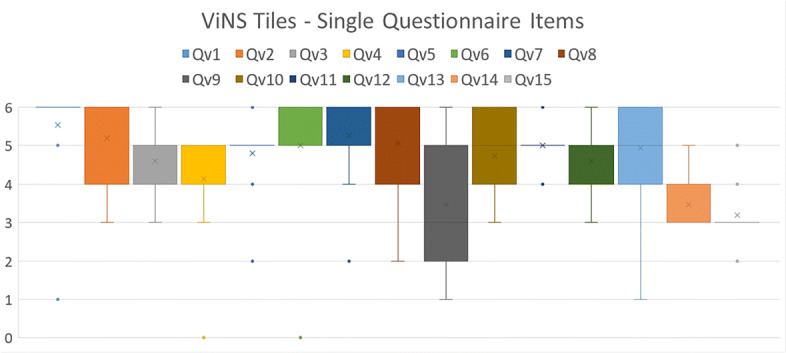
Descriptive statistics of the single items Qv1-Qv15 for ViNS Tiles using box-whiskers plots

Regarding the ViNS Tiles authoring toolkit, Fig. 18 illustrates the value distributions of the single items Qv1-Qv15 in a box-whiskers plot. It shows that the medians of all single items lie above the value of 4, except for Qv9, Qv14 and Qv15. The largest deviations are shown at Q9 and Qv13, ranging from values of 1 to 6. Qv1, Qv5, Qv11 and Qv15 present narrow box-whiskers spectra consisting of one value only, 6, 5, 5, and 3 respectively. Outliers can be observed at the items Qv1, Qv4, Qv5, Qv6, Qv7, Qv11 and Qv15. The outliers at Qv4 and Qv6 represent the lowest values of all items with a value of 0. Qv1 contains an outlier at 1.

The outcome of the single items for ViNS Tiles were aggregated to the four aspects A1-A4. The result is shown in Fig. 17 (right). Mean values for A1, A2 and A4 lie between 4 and 5. The mean value for A3 lies above 5. The box-whisker plots range from 1 to 6 for A1 and A4, 2 to 6 for A2 and 1 to 6 for A3. An outlier can only be detected for A2, located at the value of 0.

We conducted a non-parametric and independent Mann-Whitney U test [48] for each aspect A1-A4 to compare CoNMoD and ViNS Tiles. With a threshold for statistical significance of 5%, three of the four tests point out that there exist significant differences. These significant differences occurred at the aspects ease of use (A1) with *p* = 0.0271, workflow (A2) with *p* = 0.00148 and efficiency (A4) with *p* ≤ 0.00001. At all these aspects, CoNMoD received higher absolute mean values (Fig. 17). The test for comparing the clarity (A3) did not indicate a significant difference and resulted in *p* = 0.4009. The graphs of both toolkits in Fig. 17 present similar deviations of thevalues for A3.

**Fig. 19:**
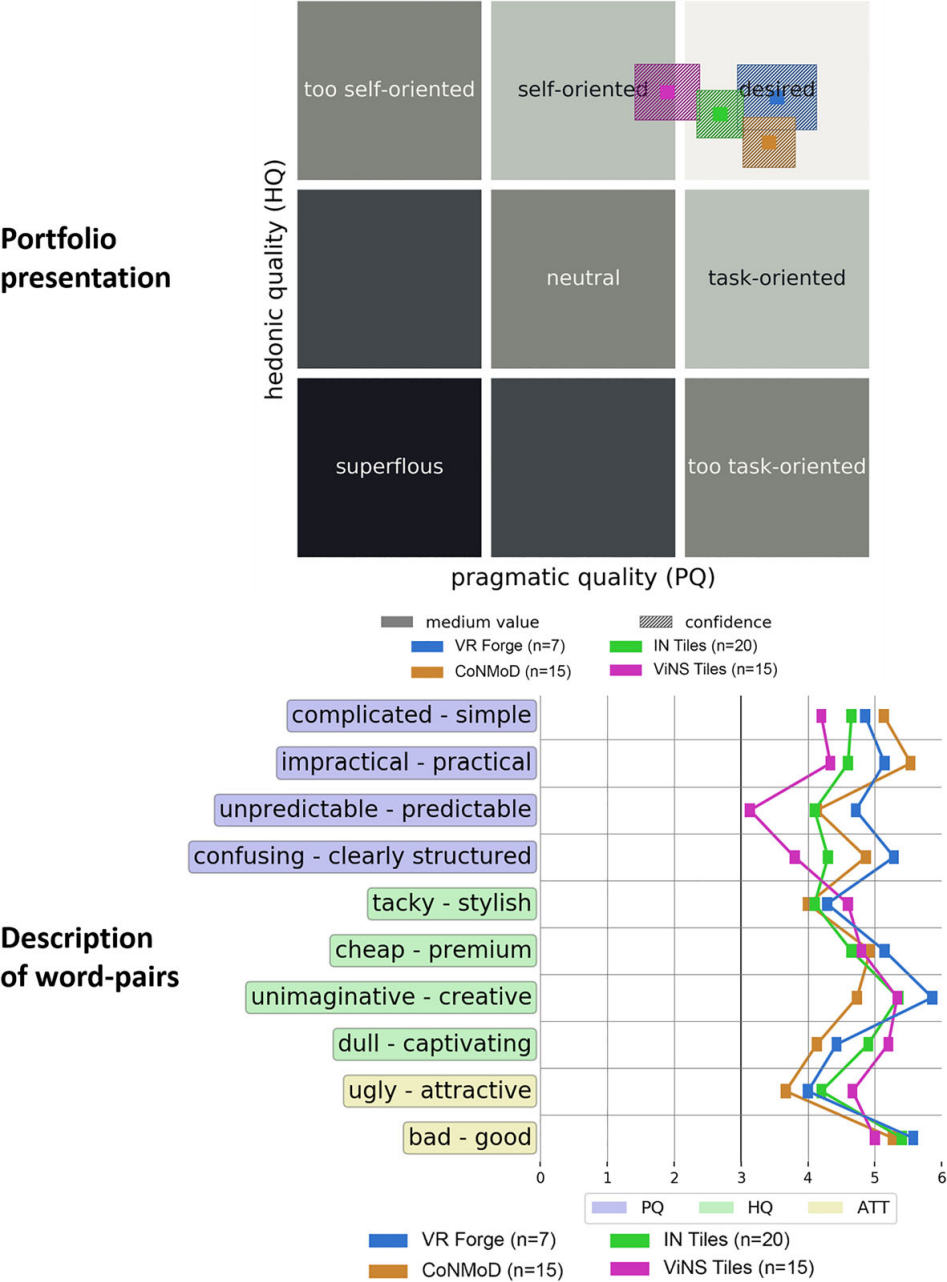
Word-pairs visualization (bottom) and portfolio-presentation (top) of the AttrakDiff’s outcome comparing all four existing VR nugget authoring toolkits: VR Forge (blue), IN Tiles (green), CoNMoD (orange), and ViNS Tiles (pink)

In addition to the above-mentioned questions and aspects, we analyzed the outcome of the AttrakDiff items. Figure 19 illustrates the results in the description of word-pairs (bottom) and the portfolio-presentation (top). The latter classifies the authoring toolkits according to their hedonic and pragmatic qualities. Furthermore, the figure illustrates a comparison of our introduced CoNMoD and ViNS Tiles toolkits with the existing VR Forge [31] and IN Tiles [33] toolkits.

The portfolio-presentation (Fig. 19 top) shows that CoNMoD is placed within the ‘desired’ area with a slight shift towards ‘task-oriented’. ViNS Tiles was classified within the ‘self-oriented’ area, whereas its according confidence rectangle overlaps with the ‘desired’ area. Compared to VR Forge and IN Tiles, the confidence of ViNS Tiles overlaps with the confidence of IN Tiles and the rectangle of CoNMoD overlaps with both VR Forge and IN Tiles. VR Forge has the highest pragmatic quality value. ViNS Tiles has the highest hedonic quality value. It is slightly higher than the one for VR Forge.

The word-pairs visualization (Fig. 19 bottom) of the AttrakDiff items gives further insight into the single items. All points for CoNMoD and ViNS Tiles lie above the neutral value of 3. All items for CoNMoD relating to the pragmatic qualities have a higher score than the corresponding items for ViNS Tiles. The other way around, the hedonic quality items for ViNS Tiles have a higher score than the ones for CoNMoD except ‘cheap-premium’, where the values are similar. Compared to the existing authoring toolkits, the graph shows similar flows for all four toolkits with slight differences. The highest mean value is shown at ‘unimaginative-creative’ for VR Forge and the lowest value can be observed at ‘unpredictable-predictable’ for ViNS Tiles. At five items, VR Forge has the highest score – two relating to the hedonic quality, two relating to the pragmatic quality and one relating to the overall attractiveness. CoNMoD holds the highest scores at two items relating to the pragmatic quality and ViNS Tiles holds three high-scores – two relating to the hedonic quality and one relating to the attractiveness. IN Tiles does not hold any high-score, but also does not hold any lowest.

We obtained the evaluation data from the VR Forge [31] and the IN Tiles evaluation [33] and conducted a Kruskal-Wallis test [42] with a threshold for statistical significance of 5% to draw conclusions on the significance of the three AttrakDiff categories’ differences between the four toolkits. The tests did confirm significant differences between the hedonic (*p* = 0.024654) and pragmatic qualities (*p* ≤ 0.00001) and none for the overall attractiveness (*p* = 0.771612). We conducted further post-hoc tests (after Conover [12]) to identify between which toolkits the differences occurred. Table 2 illustrates outcomes of this analysis.

**Table 2:** Analysis on significant differences of the product character consisting of hedonic quality

Aspect	Toolkits	Overall Ø-scores	Different from variable number
		(rounded)	(Kruskal-Wallis test with *p* ≤ 0.05)
Hedonic Quality	(1) *VR Forge*	4.9286	
	(2) *IN Tiles*	4.75	
	(3) *ViNS Tiles*	4.9833	(4)
	(4) *CoNMoD*	4.45	(3)
Pragmatic Quality	(1) *VR Forge*	5	(2), (3)
	(2) *IN Tiles*	4.4125	(1), (3), (4)
	(3) *ViNS Tiles*	3.8667	(1), (2), (4)
	(4) *CoNMoD*	4.9167	(2), (3)
Attractiveness	(1) *VR Forge*	4.7857	
	(2) *IN Tiles*	4.8	
	(3) *ViNS Tiles*	4.8333	
	(4) *CoNMoD*	4.4667	

pragmatic quality and the overall attractiveness [27, 28] between the four VR nugget authoring toolkits (VR Forge, IN Tiles, ViNS Tiles and CoNMoD). A Kruskal-Wallis test [42] with following post-hoc tests after Conover [12]

**Fig. 20:**
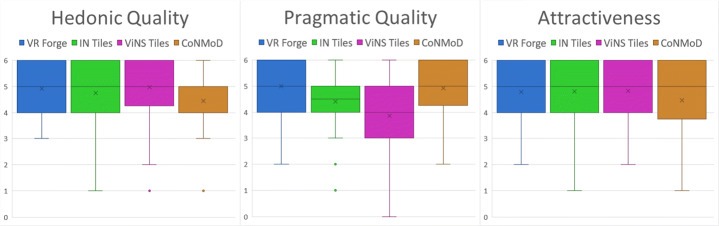
Descriptive statistics of the product character by the three aspects hedonic quality, pragmatic quality and attractiveness

Regarding the hedonic quality, the only significant difference that can be observed is located between ViNS Tiles and CoNMoD. With the highest hedonic quality score of all four toolkits, it is in favor of ViNS Tiles. The analysis of the pragmatic quality did confirm several significant differences. Both VR Forge and CoNMoD differ from IN Tiles and ViNS Tiles, whereby VR Forge and CoNMoD have higher scores. Both IN Tiles and ViNS Tiles differ from all toolkits respectively, with IN Tiles having the higher score of these two and ViNS Tiles having the lowest score. Figure 20 illustrates the hedonic quality, pragmatic quality and attractiveness values.

#### Discussion of the results

Regarding CoNMoD, our evaluation indicates the toolkit was positively perceived by our participants. Apart from outliers, only Qc4 and Qc16 show values within the box-whiskers plots that are located below the neutral value of 3. Qc4 indicates that some participants had difficulties navigating through the 3D scene with the proposed module-centered camera control. To give authors more freedom, this feature can be made optional. A free moving camera can be used to navigate through the scene, and when working on a specific module, the proposed module-lock can be activated to stay in focus of the current editing module. Except this item, the other items relating to the ease of use (A1) of the CoNMoD toolkit have high scores in mean. That indicates that our participants could easily use the toolkit. Qc16 relates to the efficiency (A4) and evaluates if our participants can achieve similar results with other software. Especially participants with higher experience of VR and 3D software stated that they could have implemented the VR nuggets with different software. This did not largely affect the overall score of the efficiency, as other items about efficiency obtained high mean scores. The items on workflow (A2) and clarity (A3) aspects show similar characteristics, so that our participants knew how create VR nuggets based on our modular toolkit without large challenges in finding certain elements on the interface or not knowing what to do next during the authoring.

Regarding the proposed ViNS Tiles authoring toolkit for VR nuggets, our report indicates that our participants could use the visual scripting approach and the tile-structures to successfully create VR nuggets. However, there occurred outliers and for three items (Qv9, Qv8, Qv13), the lower whisker of the boxplot includes values below the neutral value of 3. Item Qv9 relates to the ease of use (A1). The participants indicate that the linking of the callouts to specific places at 3D models was challenging. This specific task was performed in the editing room of the ViNS Tiles application. In the comparable IN Tiles [33] study, these editing actions were performed using immersive authoring techniques. The study indicates that this task could be performed very well in IN Tiles. The workflow (A2) was rated positively by the participants with only few outlier exceptions. Our participants understood the proposed authoring process (Qv11) and knew when and how to assemble the VR nugget tiles within the authoring room (Qv3and Qv4). At this aspect, the original IN Tiles study [33] indicates challenges within the immersive authoring toolkit. After all, both ease of use (A1) and workflow (A2) of ViNS Tiles and IN Tiles could benefit from a mixed techniques toolkit, that allows authors to assemble the tiles with our ViNS Tiles authoring room and then adjust the content within the immersive IN Tiles editing room.

Based on the outcome of the Mann-Whitney U tests, we reject the null hypotheses H1_0_, H2_0_, and H4_0_ and accept their alternative hypotheses. A direct comparison of the aspects A1-A4 of CoNMoD and ViNS Tiles shows that CoNMoD obtained higher mean scores at every aspect, so that the verified statistical significant differences of the alternative hypotheses are in favor of CoNMoD. We cannot reject H3_0_.

The comparison of all four existing authoring toolkits concerning the product character (A5) shows that all toolkits received comparable hedonic and pragmatic quality-scores, with slight deviations and outliers. Based on the result of the Kruskal-Wallis test, we reject H5_0_, since there exist statistical significant differences concerning the toolkits’ product character. The visual scripting toolkits (IN Tiles and ViNS Tiles) provided high hedonic qualities. In particular, the hedonic quality of the ViNS Tiles interface was rated significantly higher than the CoNMoD interface, that is based on direct and context-based interactions with the VR content. Regarding the pragmatic quality, VR Forge and CoNMoD offered higher practical use than the visual scripting competitors. No conclusion can be drawn about differences regarding the attractiveness of the four interfaces. The absolute values of the AttrakDiff items indicate that VR Forge was most accepted but significant differences do not point out one particular toolkit as the most suitable one.

However, all toolkits were rated positively by the participants of the four studies. Outliers and larger deviations within single aspects suggest that most participants liked the prototypes, but also that all studies included participants that have not consented to certain aspects of the individual authoring toolkits. This strengthens the importance of a standardized exchange format for VR nuggets, so that authors can freely use the toolkit of their choice and still cooperate with authors that prefer a different one. Pragmatic-oriented authors can use CoNMoD or VR Forge, whereas hedonic-motivated authors can use ViNS Tiles or IN Tiles. Authors are enabled to participate in the same community and provide novel VR nuggets or make use of existing default VR nuggets to support a sustainable VR development.

Finally, we derive the recommendations that future authoring UIs letting non-experts create short VR learning applications should build on established and concrete authoring metaphors such as utilized within VR Forge or CoNMoD instead of more abstract content representations such as in IN Tiles or ViNS Tiles. However, concerning the workflow, our study indicates that that the division of the authoring process into steps similar to the room concept of ViNS Tiles and IN Tiles can be valuable to non-expert authors. It was clearly understandable when to perform preparatory actions (*authoring room*) and when to perform more spatial related actions such as re-positioning callouts of a show and tell VR nugget (e.g., *editing room*). This suggests that future interfaces should incorporate the UI of VR Forge or CoNMoD and the workflow of IN Tiles or ViNS tiles. Furthermore, based on our study results we derive the recommendation that the process of creating short and pattern-based VR content could benefit from utilizing both immersive and non-immersive authoring technologies. For example, after preparatory actions (e.g., inputting text and 3D models, selecting a specific VR nugget pattern, adding functional coatings etc.) are performed within the *authoring room* stage using non-immerisve hardware, the UI could switch to immersive hardware for performing spatial related actions (e.g., for re positioning callouts, adjusting the size of 3D models etc.) since we could notice that our layperson authors had difficulties in both of our proposed tools with these aspects.

## Conclusion and future work

In this paper, we introduced two novel authoring toolkits that enable authors to create short pattern-based VR experiences using the VR nugget concept: *CoNMoD* provides an authoring interface that realizes the VR nugget approach based on context-related modules and *ViNS Tiles* utilizes visual scripting based on tile-like affordances to let authors assemble VR nuggets. In two separate user studies, we evaluated the introduced authoring toolkits and indicated that both were accepted well by our participants. Furthermore, we compared the proposed toolkits to two existing ones and have indicated that all four are well-suited to create VR nugget content. Slight differences regarding their hedonic and pragmatic qualities indicate that VR Forge and CoNMoD are suited for pragmatic-motivated authors whereas IN Tiles and ViNS Tiles are well-suited for hedonic-driven authors. VR Forge provides a balanced mix of both qualities but does not support *nugget authors* to create novel default nuggets.

To enable authors to use the toolkit of their individual choice and still participate in an overarching authoring community, we investigated a data structure to propose VR nugget standard. It can be used to exchange VR nuggets between different authoring toolkits and fosters the reusability of VR nuggets. Furthermore, we explored the dissemination of VR nuggets and drew conclusions about the realization of a VR nugget exchange. Our participants, as potential authors, would use our dissemination-approach and are willing to share their VR nugget realizations and contribute to a greater VR nugget authoring community.

Concepts and practical insights in the authoring of VR nuggets provided by this paper facilitates the usage of VR technology for the current microlearning paradigm within the educational sciences. We support educators to overcome VR authoring challenges and to include VR as an emerging alternate reality technology within existing curricular structures. But the VR nugget concept is not restricted to the educational domain and so is our approach for disseminating VR nuggets. VR nuggets can also be used to create small VR experiences for other domains, such as medical VR. VR nuggets for these domains can use the same standard as the ones that rely on educational patterns. For example, metadata of a VR nugget could include tags that are used to identify to which domain they belong originally to help authors find suitable VR nuggets.

With a view to limitations of our work, we already discussed different concept-related aspects such as the design of IN Tiles’s hexagonal tile affordances and the practicality of our tools helping overcoming the challenges of creating interactive real-time systems and handle content-related tasks. One limitation of the conceptual nature of VR nuggets is that we limit authors such as educators to adopt the *content author* role with our tools. It can be discussed whether content authors may also take over parts of the *nugget authors’* processes. Our concept does not intend to draw straight lines between the author roles but rather illustrates the focus of three different roles that we identified. Certainly, there are tasks that overlap or need to be done as a team effort. For example, identifying novel patterns that should be included within our authoring systems will surely benefit when content authors as domain experts from the application domain and nugget authors work close together. In that case, content authors would influence the design of a novel default VR nugget’s functionalities to a great extent. Thus, the interfaces between the roles we identified should be examined in future work. Tools that support authors working together or even take different roles within VR-nugget-based VR authoring processes could be beneficial. The roles might be further divided into *power authors* and *standard authors* within each role. For example, *power content authors* that are technically affine and have familiarized themselves with VR nuggets might be provided with separate tools that give them access to the Unity project where nugget authors integrate novel patterns as default VR nuggets. Unity allows creating custom interface tools that extend the initial editor interface of Unity. Such tools for power content authors may be developed by *system authors*. However, tools for power content authors do not have to be placed within the Unity editor itself. Separate tools may suffice for creating novel types based on the given default VR nuggets as well.

Concerning limitations of the evaluation methods, further quantitative measures besides the standardized AttrakDiff questionnaire, such as the time to complete, errors made, could give been utilized and paired with the qualitative feedback to corroborate our findings. Besides the mentioned usability measures, a heuristics evaluation could have been utilized, however, existing standardized heuristics are hardly applicable for the design of VR authoring UIs. Finally, the expert interview evaluation of our concept could have also been used for deriving requirements of our concept before the implementation and the user study.

From a technical point of view, we also revealed details are still challenging regarding the design and realization of VR authoring systems using game engines. In our example, Unity supports importing a plethora of asset formats in the game engine’s original authoring environment, such as 3D models in .fbx, .obj, .3ds, and several more formats. This compatibility is not included our authoring tools that are built with the game engine. For example, the possibility to load a 3D model at run time into an authoring tool that is not the Unity editor, but build with the Unity editor, must be programmed from scratch, as Unity does not provide such functionality by default. Still, paid plug-ins already exist covering such functionality. Our proposed systems and their components were built for supporting only the .obj format, that was used as a proof of concept and representative for other formats. For example, conceptually, formats such as FBX or glTF can also be included. Still, there exist many different 3D modeling tools and online sources providing 3D models in various formats. To support authors that are not familiar with the usage of 3D software, more 3D formats should be supported by the proposed systems. Alternatively, a pre-processing or conversion tutorial based on free 3D software such as Blender could be provided to help domain experts overcoming this technical challenge. Otherwise, the tools’ real-world use will be limited, even though first attempts to apply the authoring tools to real-world applications have shown that .obj was sufficient.

Future work relating to the contribution of this paper can be divided into two categories: Planning multimedia presentations that include VR nuggets and the actual presentation approach of them. VR nuggets are currently used to complement courses and sprinkle small VR experiences in between nuggets that are implemented with other media. While authoring software for slideshow presentation, such as PowerPoint, does already integrate various media, such as videos, animations and images, the integration of VR in common presentation technology is not established, yet. To bring VR nuggets into a temporal relation to other learning nuggets, an integration of VR nuggets into established media authoring software can support the planning process of multimedia presentations that include VR nuggets.

After a VR-mediated presentation can be planned and the VR nuggets can be authored, the presentation itself still involves challenges. Transitioning from common media to VR but also back from VR to the physical environment can help to integrate small VR experiences seamlessly in presentations. A naive integration would build nuggets as standalone VR applications, then interrupt the slide presentation and execute the VR nugget applications and vice versa. These ‘interruptions’ must be explored for creating smooth transitions from and to VR in learning sessions Furthermore, there exist several user roles relating to VR nuggets. At the example of a presentation, we can separate the author of a presentation from its actual presenter. Presenters might not need to exchange content of a VR nugget but have different requirements on a system that handles VR nuggets, such as interacting with the audience during the presentation. With our nugget exchange format, we enable a workflow that encapsulates the authoring from the presenting techniques of VR nuggets. A separate *nugget player* can be used to support the role of presenters. It can handle the execution of VR nuggets and the connection to established presentation software independently of the authoring toolkits. It can also include the transitions from and to VR nuggets, as these are not needed in an authoring toolkit. This player could be implemented as a standalone application or as a direct add-in in presentation software and could respond to the needs of presenters that use VR nuggets.
